# Naval Hygiene and Scurvy

**Published:** 1859-01

**Authors:** 


Health of the Navy.

Dr. Alexander Armstrong, surgeon to Her Majesty's ship *Investigator* during the recent polar expedition, has published a valuable work entitled *Observations on Naval Hygiene and Scurvy, more particularly as the latter appeared during a Polar Voyage.* In all previous arctic expeditions, scurvy had appeared during the first year; but, in the present instance, two years and three-quarters elapsed before the disease appeared on board the *Investigator*, notwithstanding a sea-voyage of six months duration, and a sojourn of two months on the ice,—during the first of which the crew had an insufficient supply of food, and in the second of which the allowance was reduced to two-thirds of the original quantity. Their long exemption from disease was the result of proper attention to certain hygienic measures, which ought always to be followed on board ship. These are:—care in the selection of a healthy crew, especial attention being paid, among other things, to the state of the chest, and of the hepatic and splenic regions; maintaining a pure and dry air between decks, and keeping the decks always dry; pumping the bilges daily, and washing them with salt water; sufficient clothing for the men, flannel being worn next the skin; removal of wet clothes; sea-bathing once daily; the promotion of cheerfulness,—for which a musical band would be very useful; and a proper supply both of animal and of vegetable food, and of that well-proved preventive of scurvy, lemon-juice. Regarding lemon-juice, Dr. Armstrong says that, though it may be issued regularly to the men, it does not follow that it is properly consumed:

"This I know from experience, not alone from observing the practice adopted in polar ships, but likewise from my observations made in the general service of the navy. When the lime-juice is taken into the different messes, many of the men are careless about it, and seldom or never take it; others will give it away, or, what is a common occurrence, will barter it with the few who are wise enough to prefer it to spirits,—so that one half of the crew may not in all probability take it. Thus there is no certainty whatever that it is taken by all with any degree of regularity; but there is positive evidence to the contrary. In order that each man should drink his proper allowance, I proposed for adoption the plan I have already mentioned,—which was very strictly carried out. I could wish that this plan was enforced in the navy, not only as regards lime juice, but likewise spirits; for I am quite satisfied that by making the men drink their allowance of either at the tub where it is mixed in presence of the officer who always superintends the 'grog tub', the most beneficial results would accrue, not only in a sanitary point of view, but in preventing the occurrence of drunkenness and its attendant evils on board ships of war."

